# *Pasteurella multocida* Septicemia in a Patient with Cirrhosis: An Autopsy Report

**DOI:** 10.1155/2015/597806

**Published:** 2015-01-27

**Authors:** Takuma Yamamoto, Takahiro Umehara, Takehiko Murase, Kazuya Ikematsu

**Affiliations:** Division of Forensic Pathology and Science, Nagasaki University School of Medicine, Nagasaki 852-8102, Japan

## Abstract

More people are keeping pets in their homes but may not be sufficiently aware of the potential danger from infections. We report an autopsy case of a 57-year-old man affected by cirrhosis. Septic shock with *Pasteurella multocida* pneumonia was the cause of his death. *P. multocida* was the source of infection via the respiratory tract and caused pneumonia. Cirrhosis is one of the risk factors for *P. multocida* infection. A detailed patient history about animal exposure should be obtained and a differential diagnosis of *P. multocida* infection must be kept in mind.

## 1. Introduction

In the developed world, more and more people are keeping pets in their homes and share a happy life with pets as family members. Cats are one of the most popular domestic animals but owners are not generally aware of their potential danger with respect to infectious complications. We report an autopsy case of a 57-year-old man affected by cirrhosis who developed pneumonia from* Pasteurella multocida *(*P. multocida*) infection, which resulted in rapidly fatal septic shock.

## 2. Case Presentation

A 57-year-old man had a cat in his home and often had close contact with the pet. He was a heavy drinker of alcohol. One morning, he had a high fever and fatigue from awakening. He went to work, but he could not continue working and had a rest. A few hours later, his colleague found that he lost consciousness and was sweating. Emergency services were called and he was transferred to the hospital emergency department where he died following cardiopulmonary arrest.

Laboratory tests were conducted on admission. Hematology revealed total leukocytes 2050/*μ*L, platelets 64,000/*μ*L, and hemoglobin 13.1 g/dL. Blood chemistry showed C-reactive protein 1.16 mg/dL, procalcitonin 8.24 ng/mL, albumin 2.9 g/dL, total bilirubin 1.0 mg/dL, and PT-INR 1.23. HBs-Ag and HCV-Ab were negative. Blood culture revealed a Gram-negative coccobacillus, which was identified as* P. multocida*.

**Figure 1:**
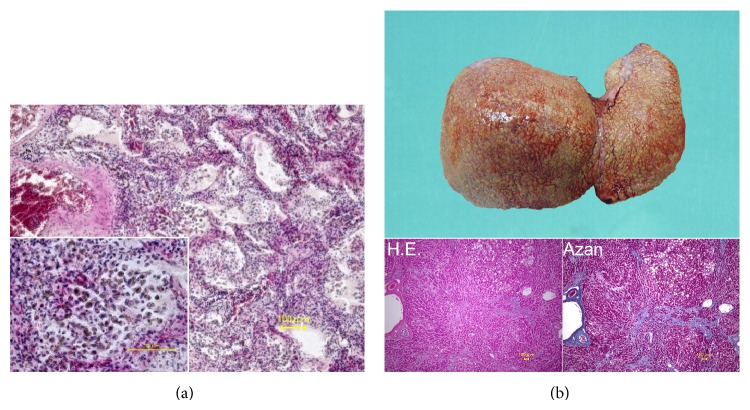
(a) Lung. Microscopic alveolar examination showing diffuse inflammatory macrophage and lymphocyte cell infiltration, suggesting pulmonary pneumonia. (b) Liver. Macroscopic examination showed liver cirrhosis. Microscopic examination showed pseudolobule in the liver (hematoxylin eosin staining and Azan staining).

Autopsy was performed on the following day. No injuries such as scratches or bite wounds were found on external examination. In addition, there was no cellulitis. On internal examination, the right and left lungs weighed 770 and 700 g, respectively. Microscopic examination showed diffuse inflammatory macrophage and lymphocyte alveolar infiltration, suggesting pulmonary pneumonia (Figure 1(a)). The liver weighed 1850 g and showed cirrhosis (Figure 1(b)). The right and left kidneys weighed 180 and 170 g, respectively. Both medullae were congestive, suggesting a pathological change consequent to shock.

The diagnosis was septic shock. The focus of infection was pneumonia due to* P. multocida*. Cirrhosis was probably due to alcohol.

## 3. Discussion

Pasteurellae are small, Gram-negative, aerobic, facultatively anaerobic, non-spore-forming coccobacilli and are known as zoonotic agents for human disease.* P. multocida*,* P. septica, P. canis, P. stomatis,* and* P. dagmatis* are the major species responsible for pasteurellosis in humans [1, 2]. Pasteurellosis is a rare zoonotic disease. However,* P. multocida* is widespread throughout the world. It is present in the normal flora in the nasopharynx and upper respiratory tract of domestic and wild animals such as cats (70–90%), dogs (50–60%), and pigs (50%) [3–6].* P. multocida* infection often results in local cellulitis and subcutaneous abscesses within skin and soft tissues following animal scratches or bites [1, 5, 7]. Direct inoculation via bites is the main cause of pasteurellosis.

In the present case, the patient had previously appeared healthy, although he had suffered from cirrhosis. There were no signs of injury such as scratches or bites on external examination, though traumatic animal contact is the most common cause of* P. multocida* infections. Macroscopic examination revealed diffuse pneumonia. The respiratory tract is one of the possible sites for* P. multocida* infections [8]. We thought that* P. multocida* caused infection via the respiratory tract, resulting in pneumonia and septic shock.

Septic shock caused by* P. multocida* bacteremia is rare with fewer than 100 cases having been reported in the literature [9]. In such cases, it is often associated with a compromised immune system resulting from diverse factors such as chronic renal failure, cancer, or cirrhosis [4]. Liver dysfunction has been reported to be a major factor for* P. multocida* bacteremia [1, 6]. The causal mechanism is believed to result from impaired reticuloendothelial function and portosystemic shunting [1, 4], as the hepatic reticuloendothelial system is an important site for removing bacteria from blood [3]. Our patient had cirrhosis, but he otherwise appeared healthy. Laboratory data did not reveal severe liver failure. Child-Pugh score was grade A. Despite such liver function, he became infected with* P. multocida*.

We would recommend that not only immunocompromised patients but also patients with cirrhosis who are otherwise apparently healthy should avoid animal contact to prevent pasteurellosis. With an increasing opportunity for contact with domestic animals, the potential for* P. multocida* infection is becoming more widespread. It is important to inform otherwise healthy patients with cirrhosis about* P. multocida* infection and the danger from animal exposure [5].

With respect to emergency medicine or postmortem examination, we should obtain a detailed patient history about animal exposure in such cases of rare infection. A differential diagnosis of* P. multocida* infection must be kept in mind and it is important to survey whether the deceased had a pet or not.
